# Fabrication and Properties of Chitosan/Calcium Polyphosphate Fibre Composite Biological Scaffold

**DOI:** 10.3390/gels11100767

**Published:** 2025-09-24

**Authors:** Xiaohu Qiang, Zhu Hu, Wang Liu, Dajian Huang

**Affiliations:** School of Materials Science and Engineering, Lanzhou Jiaotong University, Lanzhou 730070, China; qiangxh@mail.lzjtu.cn (X.Q.); 11230942@stu.lzjt.edu.cn (Z.H.); 12222085@stu.lzjt.edu.cn (W.L.)

**Keywords:** chitosan, calcium polyphosphate fibre, biological scaffold, mechanical strength

## Abstract

Natural biomaterials are widely used in the construction of cartilage tissue engineering due to their excellent biocompatibility, easy degradation, and ability to degrade products to be absorbed by the human body. However, due to their poor mechanical properties, it is usually necessary to composite them with other materials to prepare biological scaffolds that meet the expected requirements. This study used freeze-drying technology to introduce calcium polyphosphate fibres (CPPFs) into a chitosan (CS) matrix to prepare composite scaffolds with better performance. CPPF was used as a filler and inorganic skeleton in the CS/CPPF composite to improve the properties of the CS-based scaffold. With little change in porosity, the compressive strength of the CS/CPPF composite scaffold increased from 0.172 MPa of chitosan to 0.332 MPa with the increase in CPPF addition. The water absorption rate of the composite scaffold decreased from 1297.42% to 935.37%. In vitro degradation experiments revealed that CPPF accelerated the degradation of the scaffold and generated calcium phosphate and nano-hydroxyapatite compounds during the degradation process. According to our cytotoxicity testing, the CS/CPPF composite scaffolds exhibited good biocompatibility and could enhance cell proliferation. This method of incorporating CPPF into CS provides important reference values for the application of CPPF in other natural bone tissue engineering scaffold materials.

## 1. Introduction

Bone tissue engineering has provided a new alternative method for the treatment of bone defects [1,2]. As a carrier of cells and signalling factors, scaffold materials provide a unique porous structure and microenvironment for bone tissue regeneration, which is a key research issue in bone tissue engineering [3,4]. At present, significant breakthroughs have been made in research on bone tissue engineering scaffolds, but there are still many research directions that need to be continuously explored, and many problems urgently need to be solved [5,6]: (1) The first issue is the insufficient mechanical performance of the scaffold, which cannot be used for weight-bearing parts of the body in practical clinical applications and cannot meet the requirements of cartilage tissue engineering for scaffold performance. (2) The degradation rate is difficult to control and cannot be synchronized with the tissue growth rate. In addition, during the degradation process, it is easy to cause inflammation of cartilage tissue, which affects the growth and development of bones. (3) How to improve the affinity, compatibility, and biological activity of scaffold materials for tissue cells remains an issue. At present, a variety of inorganic materials, polymer materials and organic–inorganic composites have been used to prepare bone tissue scaffolds [7,8,9]. Among these materials, chitosan (CS) polysaccharide and inorganic calcium polyphosphate (CPP) have been studied extensively for preparing tissue engineering scaffolds [10,11].

CS is a polysaccharide with unique properties and extracted from crustacean shells [12]. It has been widely used in medicine, food, and cosmetics owing to its non-toxicity, antigenicity, antibacterial and antioxidant properties, biocompatibility, and biodegradability [13,14,15,16,17]. In addition, CS also has bone conduction and certain bone induction abilities, which can promote bone formation, and the degradation rate of chitosan is highly related to its deacetylation degree. The controlled deacetylation degree of chitosan is expected to match the degradation rate of the composite with the formation of new bone [18]. Currently, there are many studies on chitosan-based scaffolds. For instance, the gelatine/chitosan/polyvinyl alcohol/nano-hydroxyapatite scaffolds prepared by Ma et al. can effectively promote cell proliferation and adhesion [19]. Jiang et al. prepared a high-porosity porous chitosan nanofibre scaffold with an extracellular-matrix-like structure using chitosan nanofibres as raw material, providing a good adhesion and differentiation environment for MC3TM cells [20]. CS-based tissue engineering scaffolds have been studied by many researchers. However, their weak mechanical properties and low water resistance limit their practical application [21]. Previous works added inorganic fillers, such as hydroxyapatite and carbon nanomaterials, into the CS matrix to overcome the aforementioned shortcomings [22]. A small amount of these nanomaterials can improve the performance of CS-based scaffolds [23]. However, when they reach a certain addition amount, these inorganic nanomaterials easily agglomerate in the CS matrix, reducing the various properties of CS-based composite scaffolds. Therefore, the advantages of organic and inorganic materials cannot manifest in composite biological scaffolds. CPP is another inorganic material that is often used to prepare tissue engineering scaffolds, as it has a similar chemical composition to natural bone [24,25]. The preparation process of calcium polyphosphate powder is as follows [26]: First, calcium carbonate is added to phosphoric acid (15%) at a ratio of Ca/P 1:2. After the reaction ends, excess water is evaporated under vacuum, the resultant solid washed with ethanol, and the powder is calcined at 500 °C for 10 h and heated to 1200 °C to melt. The molten CPP is poured directly into ice water to prevent crystallization. Finally, the CPP powder is obtained by ball milling, followed by sieving. The alginate formaldehyde crosslinked chitosan/polyphosphate calcium composite scaffold prepared by Wang et al. promoted the adhesion and proliferation of meniscal chondrocytes [26]. Researchers have utilized CS and CPP to prepare composite scaffolds [27,28]. However, the morphology of CPP materials consists mostly of particles, and the CS/CPP composite is still similar to traditional organic–inorganic composite materials [28]. Therefore, looking for a degradable inorganic material that can be added in high amounts to a CS matrix and will uniformly disperse in the matrix can undoubtedly provide a new idea for research on biological scaffold materials.

**Figure 1:**
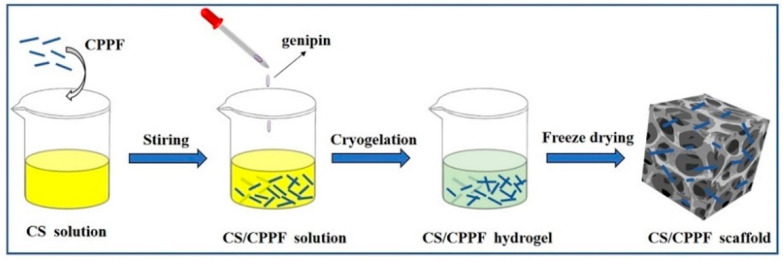
Schematic diagram of the preparation process of CS/CPPF composite scaffolds.

In previous research, we developed a type of calcium polyphosphate fibre (CPPF) with a micron-scale diameter, which has high modulus, good biodegradability, and good dispersion in polymers [29,30,31]. Thus, CPPF may be a candidate for preparing high-performance CS-based organic–inorganic composite biological scaffold materials. In this study, we prepared composite scaffolds using CPPF and CS, as shown in Figure 1. CPPF acts as the reinforcement and inorganic skeleton in the composite. We examined the effects of CPPF addition on the mechanical characteristics of the CS scaffold, the water resistance, and other characteristics of the scaffold following degradation. In addition, the biocompatibility of each scaffold was evaluated.

## 2. Results and Discussion

**Figure 2:**
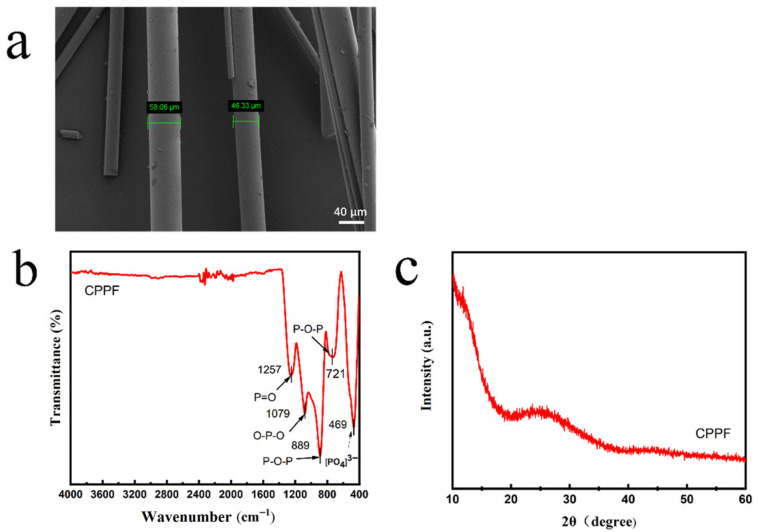
(**a**) SEM micrograph of CPPF, (**b**) FTIR spectrum of CPPF, (**c**) XRD of CPPF.

Figure 2a shows that the surface of CPPF is smooth and presents a slender fibrous morphology with a diameter of approximately 50 μm. The FTIR spectrum (Figure 2b) of CPPF shows that significant absorption peaks were observed at 1257 cm^−1^, 1079 cm^−1^, 889 cm^−1^, 721 cm^−1^, and 469 cm^−1^, corresponding to the stretching vibration peak of P=O, the absorption vibration peak of O-P-O, the asymmetric stretching vibration absorption peak of P-O-P, the symmetric stretching vibration peak of P-O-P, and the bending vibration peak of [PO4]^3−^ [32], respectively. Figure 2c shows the XRD patterns of the CPPF. The XRD pattern shows no distinct characteristic peaks, indicating that CPPF is an amorphous material.

**Figure 3:**
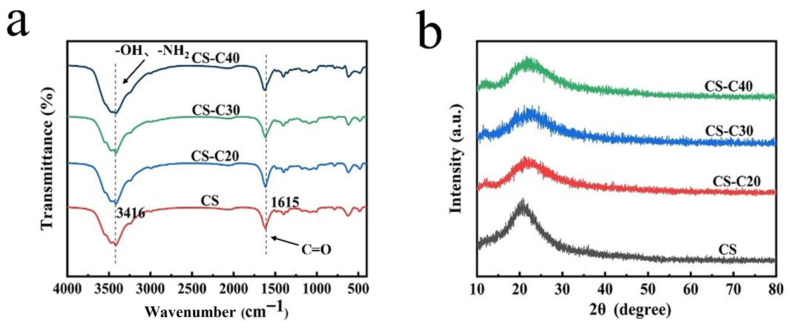
(**a**) FTIR spectrum of CS/CPPF composites, (**b**) XRD of CS/CPPF composites.

The FTIR spectra (Figure 3a) of the samples show that in the infrared spectrum of CS, the absorption peaks of 3434, 2925, and 1615 cm^−1^ are, respectively, attributed to the absorption peaks formed by –OH and N–H stretching vibrations [33,34], the asymmetric stretching vibrations of –CH_3_ and –CH_2_, and the C=O stretching of N–H (amide Ⅱ band) [35,36]. No remarkable new characteristic peaks were observed after the introduction of CPPF, possibly because CPPF was not involved in the chemical reactions. Figure 3b shows the XRD patterns of the scaffolds. CPPF and CS are amorphous materials [37,38]; thus, the XRD patterns of all CS and CS/CPPF scaffolds show only an amorphous diffraction peak of about 2θ = 21°.

**Figure 4:**
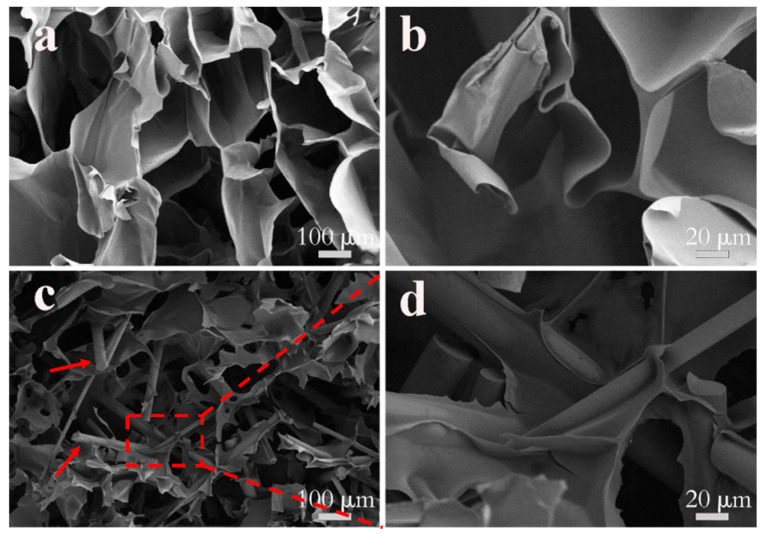
SEM micrographs of CS ((**a**) Mag = 80×; (**b**) Mag = 300×) and CS-C40 ((**c**) Mag = 80×; (**d**) Mag = 300×).

Figure 4 shows the SEM images of pure CS and CS-C40 composite scaffolds. The pure CS biological scaffold has a 3D network structure, ordered macropores, and an ordered millimetre-scale thin-sheet structure with homogeneous surfaces. This structure provides an ideal environment for cell adhesion, migration, and proliferation [39]. In the composite scaffolds, CPPF can be clearly observed, as indicated by the red arrows. CPPF shows two forms in the CS/CPPF composite scaffold: first, as fibres embedded within the CS walls, and second, as fibres inserted into the 3D network of the CS matrix, forming a reinforcing skeletal framework. In addition, the surfaces of CPPF are covered with CS, indicating good compatibility between CS and CPPF. Compared to CPP scaffolds [40], CPPF is uniformly dispersed in the CS/CPPF scaffolds without any agglomeration. These behaviours establish a foundation for the enhancement of the mechanical properties of CS-based composite scaffolds.

**Figure 5:**
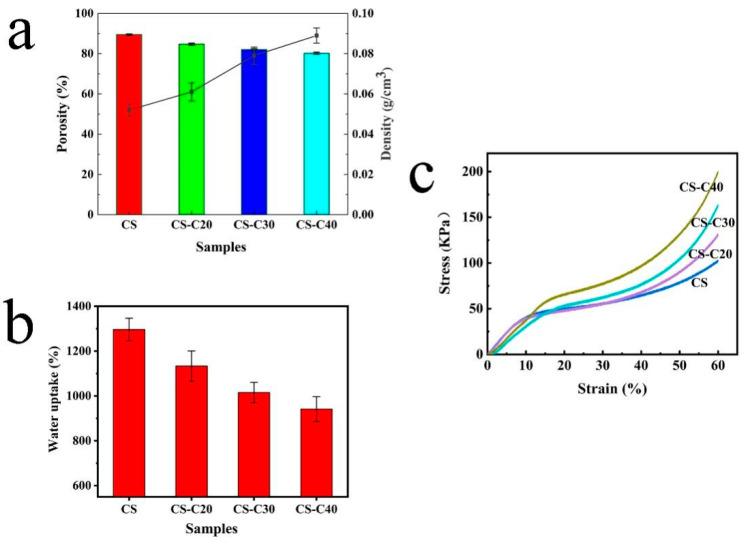
(**a**) Porosity and density, (**b**) water uptake, (**c**) compression stress–strain curves of CS/CPPF composites.

Figure 5a shows the densities of the composite scaffolds. The density of the pure CS scaffold was 0.052 g/cm^3^, whereas the density of the CS/CPPF scaffolds increased with the increase in CPPF contents. When the CPPF content reached 40%, the density increased to 0.089 g/cm^3^. This increase is due to the higher density of inorganic CPPF compared with that of CS. Another important parameter of biological scaffolds for tissue engineering applications is porosity [41,42,43]. The porosity of the composite biological scaffold is also shown in Figure 5a. The porosity of the pure CS biological scaffold was 89.45%. When CPPF was introduced, the porosity of the CS/CPPF scaffold decreased but remained high. The CS-C40 sample still has 80.22% porosity, which provides a suitable place for cell adhesion and growth.

Mechanical properties are critical parameters of biological scaffolds [42]. Figure 5c shows the stress–strain curve of the samples (no water content). The mechanical properties of the scaffolds improved considerably when CPPF was introduced into the CS matrix. The compressive strength of the CS/CPPF composite scaffolds were clearly higher than that of pure CS at each addition amount of CPPF from 20 wt.% to 40 wt.%. The compressive strength of CS-C40 (0.332 MPa) increased by 93.02% compared with that of CS (0.172 MPa). Compared with the controllable microchannel chitosan scaffold (0.15 MPa) prepared by Jiang et al. [20], CS/CPPF scaffolds exhibit better compressive strength. This increase may have occurred because CPPF and CS can bind tightly to fix the entire scaffold framework, forming a homogeneous mixture, which can be seen from the SEM images. In addition, inorganic CPPF has higher mechanical strength than CS does, thereby influencing the mechanical strengthening of composite biological scaffolds. Furthermore, the decrease in the porosity of the composite scaffold with CPPF addition contributed to the increase in compressive strength.

The water resistance of a natural polymer biological scaffold material is an important factor that affects its practical application [44]. As shown in Figure 5b, the water resistance of CS was poor, and the value of water uptake was 1297.42%, which is attributed to the high hydrophilicity of CS. In comparison, the water uptake of the CS/CPPF composite scaffolds decreased to 935.37% with the increase in CPPF content. In the case of a small decrease in porosity, the water absorption rate of composite scaffolds showed a significant decrease, which can be explained with the following reasons: First, although the addition of CPPF occupies some of the original pores, it changes the self-assembly behaviour of CS molecules during freeze-drying, causing slight adjustments to the chitosan network structure and forming new and smaller pores. The total volume of the pores may not change much, but the morphology and distribution of the pores undergo significant changes. Second, the addition of fibres may block some of the originally connected large pores, hindering the entry of water molecules. In addition, the addition of fibres restricts the movement of CS molecular chains, reduces the contact between hydrophilic groups and water molecules in the CS matrix, and thus lowers the water absorption rate. These behaviours indicate that the introduction of this inorganic framework can improve the water stability of CS-based composite biological scaffolds and further improve their practical application in biological materials.

**Figure 6:**
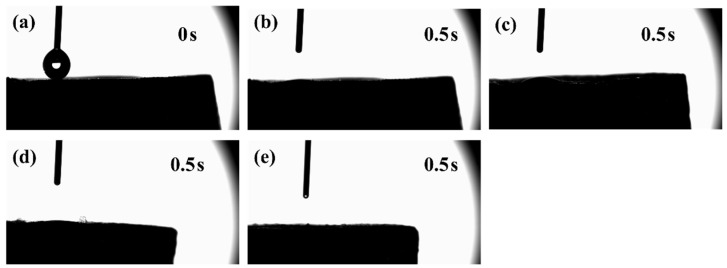
Contact angles of composite scaffolds: (**a**) the droplet image at 0 s, (**b**) CS, (**c**) CS-C20, (**d**) CS-C30, (**e**) CS-C40.

The water contact angles of the composite scaffolds are shown in Figure 6. All the scaffolds showed superhydrophilicity, and the water droplets were absorbed by the samples within 0.5 s. This phenomenon can be attributed to the highly porous aerogel structure and the intrinsically hydrophilic nature of the chitosan matrix. Numerous studies have confirmed that rough and hydrophilic surfaces are more conducive to the adhesion and growth of osteoblasts [45,46,47,48]. This property suggests that the CS/CPPF composite scaffolds may provide a favourable surface for cell attachment.

**Figure 7:**
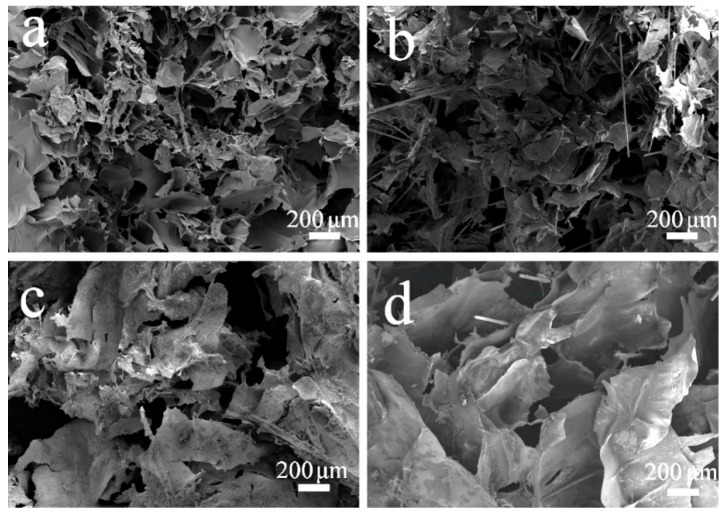
SEM pictures showing the cross-section of the CS-C40 scaffold during degradation ((**a**) after 0 weeks, (**b**) after 4 weeks, (**c**) after 8 weeks, (**d**) after 12 weeks).

**Figure 8:**
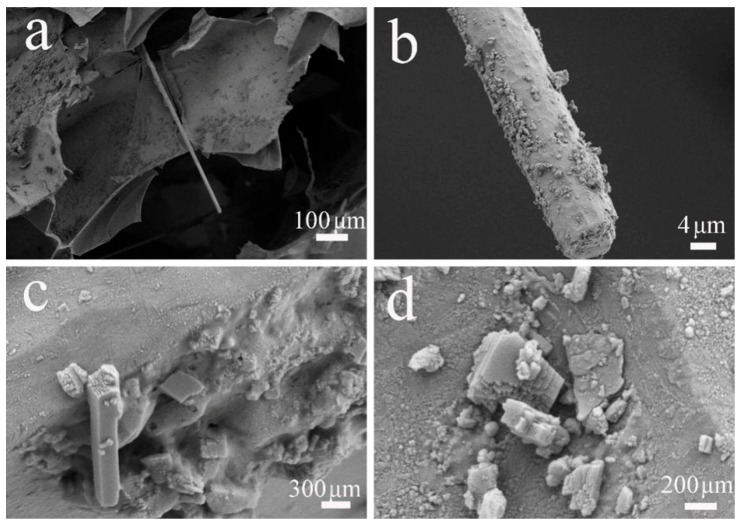
SEM images of CS-C40 scaffold after 12 weeks of degradation at different magnifications ((**a**) Mag = 80×, (**b**) Mag = 2000×, (**c**) Mag = 25×, (**d**) Mag = 50×).

The SEM images in Figure 7 show that numerous flocculent materials appeared on the smooth surface of the CS scaffolds during the degradation process, along with the phenomena of surface layer detachment and fracture. These findings suggest that CPPF has good degradability and great potential for use in the field of biological scaffolds [49]. As shown in Figure 8, the scaffold still showed good structural stability, and CPPF and CS remained rigidly attached throughout the degradation process without separation. High-magnification SEM images (Figure 8c,d) reveal that numerous crystalline deposits formed on the CPPF during the degradation process. These crystalline products are likely calcium phosphate compounds and hydroxyapatite (HA), which precipitated onto the scaffold surface as degradation byproducts of CPPF.

**Figure 9:**
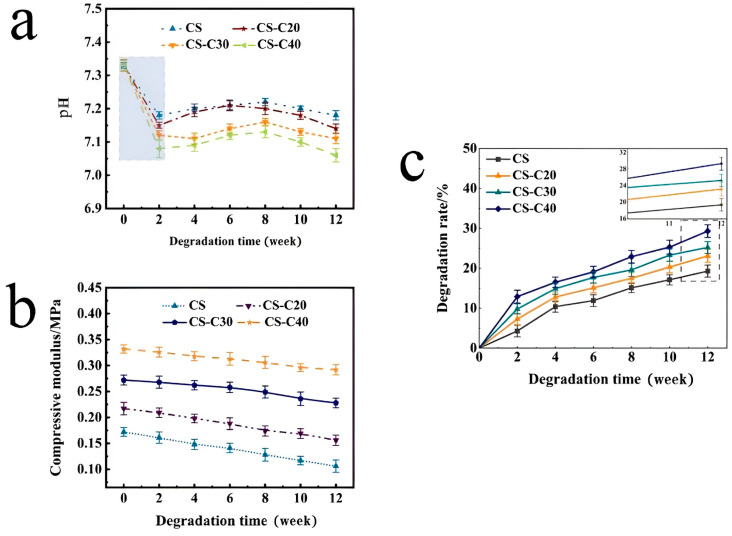
Changes in different scaffolds in PBS during the degradation process: (**a**) pH, (**b**) modulus of compression, (**c**) degradation rate.

Figure 9a illustrates the pH variation over the 12-week degradation period in PBS. Upon the addition of CPPF, the pH of the composite scaffolds in the PBS was lower than that of CS scaffolds. With an increase in the quantity of added CPPF, the pH of the scaffolds in the PBS gradually decreased, dropping from 7.20 to 7.06, which indicates a clear downward trend. This decrease may be attributed to the formation of acidic PO_4_^3−^ ions during the degradation of CPPF, resulting in an acidic solution [49,50]. Additionally, as the amount of CPPF increased, the pH of the solution decreased further. It was theorized that the degradation of CPPF was most rapid in the initial stage, followed by more stable degradation in the later stage. As the scaffold degraded, its mechanical characteristics (samples without water content) changed, as shown in Figure 9b. The CS scaffold’s compression modulus was 0.17 MPa before degradation; adding CPPF at 20%, 30%, and 40% raised it to 0.22, 0.27, and 0.33 MPa, respectively. Following a 12-week period of degradation, the compressive modulus of the scaffold dropped to 0.11, 0.16, 0.23, and 0.29 MPa. The experimental results demonstrated a relationship between the introduction of CPPF and the rate at which the mechanical properties of the scaffolds degraded. In particular, the mechanical properties of the bioscaffolds decreased more slowly during the degradation process as CPPF was added, and their mechanical properties were superior to those of the CS scaffolds. This condition was explained by the fact that CPPF was always present throughout the scaffold during its deterioration, preserving the scaffold’s overall structural integrity and maintaining the fixing effect on the scaffold. The scaffold’s changing degradation rate as the degradation time increases is depicted in Figure 9c. After 12 weeks of degradation, the CS scaffold degraded at a rate of 19.3%, whereas the composite scaffolds CS-C20, CS-C30, and CS-C40 degraded at rates of 23.1%, 25.2%, and 29.3%, respectively. The composite scaffold degraded more quickly in the early stages and at a more moderate speed in the later stages. The addition of CPPF accelerated this rate of degradation. This acceleration occurs because CS is soluble in acidic solutions and degrades more quickly in acidic environments, and CPPF creates the acid radical ion PO_4_^3−^ during the degradation process, which makes the solution acidic [49,50]. Thus, adding a certain quantity of CPPF can increase the deterioration rate of the scaffolds.

**Figure 10:**
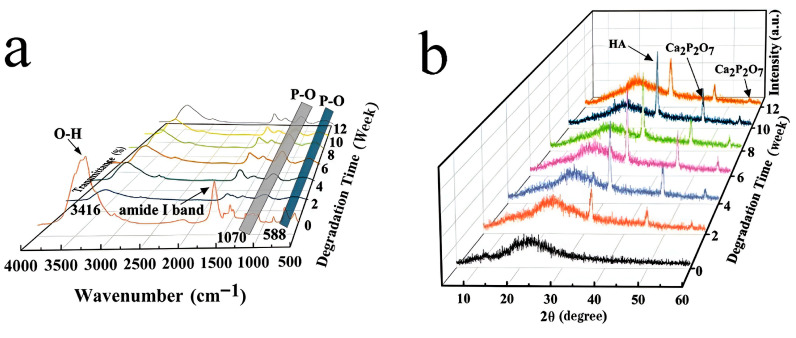
Changes in CS-C40 composite scaffolds after degradation in PBS: (**a**) FTIR, (**b**) XRD.

The FTIR spectra of CS/CPPF composite scaffolds containing 40% CPPF, degraded in PBS for various time periods, are displayed in Figure 10a. The spectra illustrates that the -OH stretching vibration peak and the N-H stretching vibration peak appeared at approximately 3416 cm^−1^, with the amide I band of N-H being attributed to 1616 cm^−1^. The intensity of the peaks significantly decreased as the degradation time increased, and these phenomena were attributed to the degradation of chitosan [51]. After degradation, P-O peaks were observed at approximately 1070 and 588 cm^−1^. With longer degradation time, the intensity of these P-O peaks increased progressively [52]. Following immersion in PBS, the phase composition of the composite scaffolds underwent significant changes, as revealed by the XRD patterns in Figure 10b. Compared to the pre-degradation state, new diffraction peaks emerged at approximately 32° and 46° in the degraded scaffolds, corresponding to hydroxyapatite (HA) and calcium pyrophosphate (Ca_2_P_2_O_7_) [52,53]. This finding suggests that hydroxyapatite and calcium phosphorus compounds were formed during the degradation of the scaffold.

**Figure 11:**
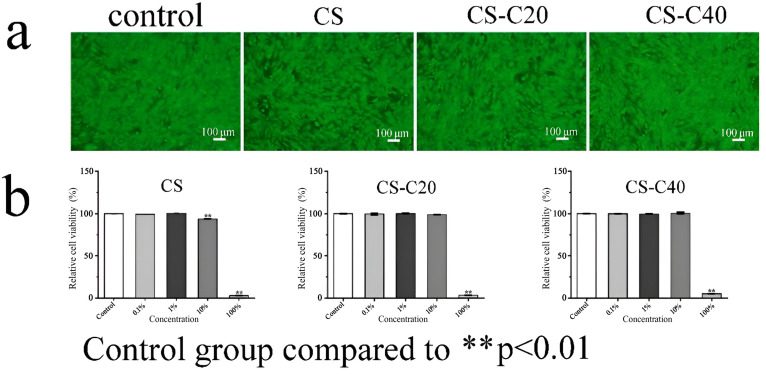
Biological properties after 7 days of incubation with different scaffold extracts: (**a**) live and dead cell staining; (**b**) relative cellular activity. *p* < 0.01 indicates a highly significant difference, marked with **.

The biological performance of each scaffold was evaluated by culturing MC3T3-E1 cells in scaffold extracts. After 7 days of incubation, the cells were examined under a fluorescence microscope, revealing minimal cell death, indicating that the scaffolds demonstrated good biocompatibility (Figure 11a) [53,54,55]. Cell viability was assessed using the CCK-8 assay, revealing that the cells exhibited high activity at extract concentrations of 0.1%, 1%, and 10% in comparison to the control group (Figure 11b). The addition of CPPF led to the formation of nano-hydroxyapatite and calcium phosphorus compounds during the degradation process, which can enhance cellular activity [56]. Findings show that the scaffold material was non-toxic and that the cells could proliferate efficiently [57].

## 3. Conclusions

In this study, we designed and prepared a new type of CS/CPPF composite scaffold using a simple freeze-drying method. The obtained CS/CPPF composite scaffolds have porous structures, higher mechanical strengths, and better structural stability in water than pure CS scaffold does. Incorporating 40 wt.% CPPF inside the CS matrix remarkably increased the compression strength up to 0.332 ± 0.09 MPa, which increased by 93.02% compared with that of pure CS (0.172 MPa). Based on the degradation behaviour of CS/CPPF scaffolds, we conclude that the incorporation of CPPF slowed the decline of mechanical properties during degradation, indicating that CPPF plays a crucial role in preserving mechanical performance post-degradation. Cell viability experiments showed that the CS/CPPF composite scaffold has good biocompatibility, and the nano-hydroxyapatite and calcium phosphate compounds produced by CPPF during degradation can enhance the activity of MC3T3-1 cells.

Compared with traditional CPP particles, CPPF exhibits superior dispersibility within scaffold materials, and as an inorganic framework, it more effectively enhances the scaffolds’ mechanical properties. This study lays a foundation for applying CPPF to natural biomaterials in bone tissue engineering and proposes novel strategies for developing related biomaterials. Subsequent research could focus on coating or grafting bioactive molecules onto the CPPF surface to improve its resistance to degradation and promote specific cell adhesion.

## 4. Materials and Methods

### 4.1. Materials

CS (with an MW of about 4.2 × 10^5^ and a degree of deacetylation of 85%, Golden-Shell Pharmaceutical Co., Ltd., Zhejiang, China). Calcium dihydrogen phosphate (Sinopharm Chemical Reagent Co., Ltd., Shanghai, China). Acetic acid (Sinopharm Chemical Reagent Co., Shanghai, China). Genipin (95%, Zhixin Biotechnology Co., Ltd., Nanjing, China). MC3T3-E1 (Suzhou Haixing Biotechnology Co., Ltd., Suzhou, China). Phosphate-buffered saline (pH 7.4 basic, Bickman Biology, Changsha, China). Cell counting kit-8 (Biosharp Life Sciences, Shanghai, China). Calcein-AM (Wuhan Sevier Biotechnology Co., Ltd., Wuhan, China).

### 4.2. Sample Preparation

#### 4.2.1. Preparation of CPPF

CPPF was prepared from calcium dihydrogen phosphate powder by high-temperature (1200 °C) melting and drawing, and the prepared CPPF was cut into short fibres of 3 to 4 millimetres using scissors for subsequent use.

#### 4.2.2. Preparation of CS/CPPF Composite Scaffold

CS was dissolved in acetic acid solution (1 wt.%) to obtain 3% CS solution. CPPF with different loadings (0%, 20%, 30%, and 40%) in CS/CPPF composites was added to the prepared CS solution, and the mixtures were stirred to obtain homogeneous mixtures. The mixtures were then crosslinked with 0.5 wt.% genipin solution (M_CS_:M_genipin_ = 6:1). Each mixture was then poured into moulds and freeze-dried (SCIENTZ-10/A, Ningbo Xinzhi Biotechnology Co., Ltd., Ningbo, China). The freeze-dried samples were named CS, CS-C20, CS-C30, and CS-C40 based on their CPPF contents.

### 4.3. Characterization

#### 4.3.1. XRD Analysis of Scaffolding

Samples were analysed using X-ray diffractometry (XRD-7000, Shimadzu, Kyoto, Japan) over a diffraction angle (2θ) range from 10° to 80°.

#### 4.3.2. Micro-Morphological Observation of Scaffolding

The microscopic morphology of the samples was observed by field-emission scanning electron microscopy (JEOL JSM-6701F, Japan Electron Optics Ltd., Tokyo, Japan).

#### 4.3.3. Fourier Infrared Analysis of Scaffolding

Fourier transform infrared (FTIR) spectra were obtained on an FTIR spectrometer (Thermo Nicolet 6700, Thermo Fisher, Waltham, MA, USA). The spectrum widths were in the range of 4000–400 cm^−1^.

#### 4.3.4. Analysis of the Mechanical Properties of Scaffolding

The compressive strength of the samples (no water content) was measured by a material testing machine (AG-IS, Shimadzu, Kyoto, Japan) with a crosshead speed of 1 mm/min.

#### 4.3.5. Water Contact Angle Testing

We used a contact angle tester (OCA25, Dataphysics, Raiffeisenstrasse, Germany) to test the contact angle of the scaffolding. First, place the sample on the stage, then drop deionized water onto the surface of the sample, wait for 0.5 s, and then measure it with the measuring instrument.

#### 4.3.6. Scaffold Porosity and Density Testing

According to the method of Zhang et al. [58], the porosity of the samples was studied and calculated by using the ethanol replacement method. The original mass of the samples was weighed (W_0_), and then the samples were sealed in a measuring cylinder containing anhydrous ethanol to prevent ethanol volatilization. After balancing for 24 h, the anhydrous ethanol was completely immersed into the inner pores of the samples, and then it was taken out and weighed, denoted as W_1_. The porosity of the samples was calculated by Formula (1).

Porosity = (W_1_ − W_0_)/(ρV_0_) (1)

where V_0_ is the volume of the samples, and V_1_ is the volume occupied by the micropores of the samples; the volume of the samples is calculated by a vernier calliper. ρ is the density of anhydrous ethanol, which is 0.798 g·cm^−3^.

According to the above experimental data, the density of the scaffold was calculated by Formula (2).

Density = W_1_/(V_1_ − V_0_) (2)

#### 4.3.7. Testing of Water Absorption of Scaffolding

Make slight modifications according to the method of Zhang et al. [58] to determine the water uptake of the samples by completely immerging the samples in phosphate-buffered solution (PBS). The water uptake can be calculated by the change in the mass of the samples before and after immersing the samples in PBS, which can be calculated by Formula (3).

Water uptake = (W_1_ − W_0_)/W_0_ × 100% (3)

#### 4.3.8. Testing of Scaffold Degradation Rates

The calculation of the degradation rate referred to Ma’s method [19]. The produced samples’ in vitro degradation performance was investigated by soaking the scaffolds in PBS for an extended amount of time and measuring the difference in scaffold mass before and after degradation. The precise procedure was as follows: The samples’ initial mass was precisely weighed and recorded as W_0_. For in vitro degradation, the samples were placed in test tubes with PBS, and each group was parallelized five times. The PBS was then replaced every seven days while the tubes were kept in a 37 °C constant-temperature biochemical incubator. After this, a batch of samples were removed periodically and washed frequently in distilled water to remove the phosphate solution, and freeze-vacuum drying was conducted. The degradation rate was then computed.

Degradation rate = (W_1_ − W_0_)/W_0_ (4)

where W_0_ represents the scaffold’s original mass, and W_1_ represents the scaffold’s mass following degradation.

#### 4.3.9. Cell Viability Testing

Referring to the method of Zhang et al. [58], MC3T3-E1 cells were taken in the logarithmic growth phase, and we counted their concentration, which was then adjusted, and they were inoculated into 96-well plates at a density of 4 × 103 cells/well with 5% CO_2_. Afterwards, the cells were incubated for the entire night at 37 °C in an incubator set at a constant temperature. The cells were then separated into four groups: CS, CC2, CS4, and control. A total of 100 μL/well of complete medium was administered to the control group; 100 μL/well of the sample extract at concentrations of 0.1%, 1%, 10%, and 100% was added to the CS, CC2, and CS4 groups. For every treatment group, three replicate wells were created, and the incubation process lasted for 7 days. The culture medium was taken out. Afterwards, 100 μL/well of media containing 10% CCK-8 was added. The optical density (OD) values were measured at 450 nm using a microplate reader (DNM-9602) to evaluate the viability of the cells.

Cell Viability (%) = (A_S_ − A_O_)/(A_C_ − A_O_) (5)

where A_S_ is the experimental group’s absorbance value. A_O_ is the background absorbance value for the A_C_ control group.

#### 4.3.10. Live/Dead Cell Staining Analysis

Logarithmic-growth-phase MC3T3-E1 cells were obtained and counted, and then the concentration was adjusted, and they were inoculated into confocal dishes at 4 × 10^4^ cells/well and 5% CO_2_. Next, the cells were cultured for an entire night at 37 °C in an incubator set at a constant temperature. The cells were cultured for 7 days after being treated in accordance with the aforementioned groupings. PBS was used to wash the cells once to remove extra serum. After 1 mL of staining solution was added per well, the mixture was allowed to sit at room temperature and shielded from light for 15 min. Three PBS washes ended the staining process, and the outcomes were noted.

## Data Availability

The original contributions presented in this study are included in the article. Further inquiries can be directed to the corresponding authors.
